# In vivo quantification of collagen-induced arthritis mouse model by three-dimensional volumetric ultrasound and color Doppler

**DOI:** 10.1371/journal.pone.0321124

**Published:** 2025-04-07

**Authors:** Qingyun Jia, Hao Xu, Tengteng Wang, Qianqian Liang, Lisheng Wu

**Affiliations:** 1 Department of orthopedics, Linyi City People Hospital, Linyi, Shandong, China; 2 Institute of Spine, Shanghai University of Traditional Chinese Medicine, Shanghai, China; 3 Longhua Hospital, Shanghai University of Traditional Chinese Medicine, Shanghai, China; University of Roehampton - Whitelands College, UNITED KINGDOM OF GREAT BRITAIN AND NORTHERN IRELAND

## Abstract

**Purpose:**

Ultrasound combined with Doppler techniques has been widely utilized to evaluate joint inflammation and internal structural changes in animal models of rheumatoid arthritis. However, previous assessments using these techniques were predominantly semi-quantitative, which may limit the precision and reliability of the results. Therefore, the primary objective of this study was to explore the potential advantages of three-dimensional (3D) volumetric ultrasound and color Doppler in quantitatively assessing the progression of disease.

**Objective:**

To quantify the severity and progression of collagen-induced arthritis (CIA) mouse model using a micro-imaging tool, 3D volumetric ultrasound and color Doppler, and assess if 3D model-based volumetric changes of joint space and vascularity correlate with clinical, histological and bone destruction findings.

**Methods:**

CIA was induced in mice on a DBA/1J background at 7 ~ 8 weeks of age. 3D volumetric ultrasound and color Doppler analysis was performed on knee and ankle joints of all mice using the Vevo 2100 system at 0, 2, 4, 8 weeks after the booster immunization. Clinical, histological and bone destruction analysis were performed as usual. Correlation analysis of the volumetric changes of joint space and vascularity with clinical, histological and bone destruction score were assessed via Spearman’s test.

**Results:**

It was possible to quantify the severity of joint inflammation and intra-articular changes during the progression of CIA by 3D volumetric ultrasound and color Doppler. The 3D model-based volumetric changes of joint space and vascularity have significant correlations with clinical, histological and bone destruction score of arthritis.

**Conclusions:**

We demonstrated that 3D volumetric ultrasound and color Doppler is a noninvasive, quantitative tool for evaluating CIA mice in vivo. Despite certain limitations, those technology significantly enhance our ability to monitor disease progression and severity, assess therapeutic interventions, and reduce reliance on invasive techniques.

## Introduction

Rheumatoid arthritis (RA) is a chronic inflammatory disorder affecting multiple synovial joints, which can cause cartilage and bone damage as well as disability [1]. Early diagnosis of rheumatoid arthritis and initiation of therapy are essential in reducing inflammation, thereby limiting disease progression, joint damage and loss of function [2]. Thus, accurate, sensitive and non-invasive imaging techniques for diagnosis of early RA are important.

During the last decade, ultrasound (US) has become an established imaging technique for diagnosis and follow-up therapeutic monitoring in patients with rheumatic diseases [3–7]. Compared to other musculoskeletal imaging modalities, US has several remarkable advantages, real-time imaging, accessibility, cost-efficiency, and absence of ionizing radiation, noninvasive, and relatively inexpensive [8,9]. Ultrasound combined with Doppler technique allows us to more accurately assess joints inflammation and changes in internal structure of joints [10–20]. Previous studies have already reported that US is more sensitive than conventional radiography in the detection of erosions in target rheumatoid arthritis joints [9,12,21], typically in the hands and feet [22–26]. Color Doppler (CD) and power Doppler (PD) modes can detect synovial hyperemia thereby predicting active synovitis and flares, and provide a semi-quantitative basis for clinical practice.

With the successful application of US in RA patients, it is also used in small animal models of RA. Recent studies have shown that US is a reliable method for evaluating the severity and progression in CIA and TNF transgenic mouse model [14,27,28]. However, those studies showed the semi-quantifiable grades or lacking of correlations of US outcomes with joints inflammation and active synovitis [28], which may lead to inaccurate results. Therefore, the development of a new, accurate and quantitative US-based detection methods for the evaluation of joint inflammatory and structural changes remains an important unmet need.

In view of these considerations, we examined the potential use of 3D volumetric ultrasound and color Doppler micro-imaging as a diagnosis tool to assess volumetric changes of joint space and vascularity in CIA mice, an established model for RA [29–31]. The results of this study demonstrate that 3D volumetric ultrasound and color Doppler can be used to quantify the severity of joint inflammation and intra-articular changes during the progression of CIA. In addition, the 3D model-based volumetric changes of joint space and vascularity have significant correlations with clinical, histological and bone destruction score of arthritis.

## Materials and methods

### Animals

Male DBA/1J mice (7 ~ 8-week-old of age) were purchased from the Shanghai SLAC Laboratory Animal Co Ltd (Shanghai, China), and housed in a laminar flow cabinet with free access to feed and water. A total of 36 mice were used for this study, and the mice were randomly divided into 2 groups, CIA modeling group and control group. All injections and imagings were performed under isoflurane anesthesia. At the endpoint of the experiments mice were euthanized with controlled flow-rate carbon dioxide.All animals received the opioide analgesic Buprenorphin (0.1 mg/kg) to prevent pain after CIA induction.

### Ethical approval

The experiments were approved by the Linyi People’s Hospital Animal Ethics Committee. The methods applied in this study were carried out in accordance with the approved guidelines and regulations.

### Induction of collagen-induced arthritis

CIA model was conducted according to a previous protocol [31]. Brieﬂy, the mice were immunized intradermally by 100 mg of bovine type II collagen (CII; Chondrex, USA) emulsified in complete Freund’s adjuvant (CFA, Chondrex, USA). To ensure a high incidence of RA induction in the CIA model, a booster immunization of chicken type II collagen emulsified in incomplete Freund’s adjuvant (IFA, Chondrex, USA) was used 21 days after the primary immunization. Typically, the first signs of arthritis appear in this model 3–5 days after the booster immunization.

### Clinical assessment of arthritis

Before the ultrasound and color Doppler scan, at each time point, the severity of the mice was monitored and scored every five days in a blinded manner for signs of arthritis in their four paws. Briefly, 0 = normal paw; 1 = slight swelling and/or erythema; 2 = pronounced swelling; 3 = ankylosis [32].

### Ultrasound assessment

**Fig 1:**
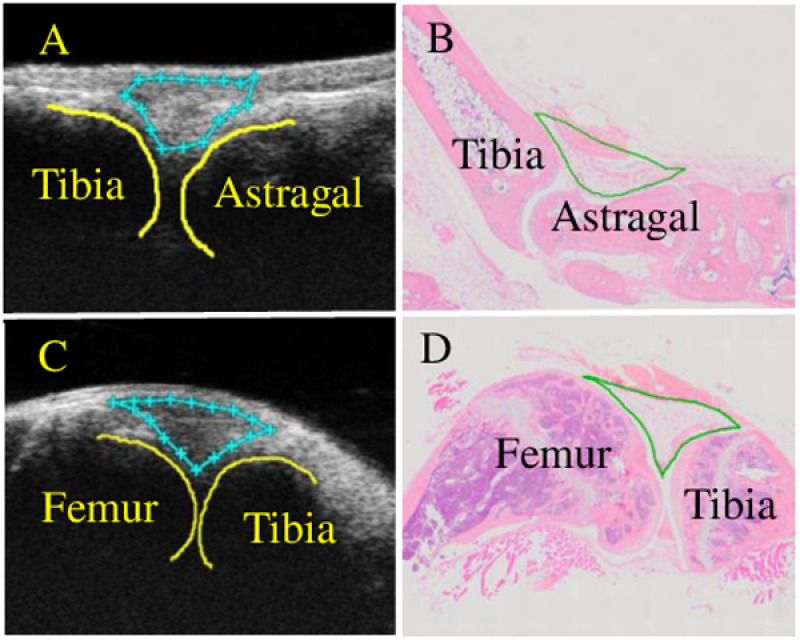
Schematic illustrations of ultrasound detection of joints space. Ultrasound images of ankle and knee joint of a mouse, and the outlined joint space, tibia, femur and astragalus (A, C). Hematoxylin and eosin (H & E)-stained ankle and knee sections and the outlined joint space, which correspond to the ultrasound detection (B, D).

At 0, 2, 4, 8 weeks after the booster immunization, the arthritis of the knee and ankle joints of all mice were visualized in a double-blind manner using the Vevo 2100 imaging system (VevoLab, FUJIFILM Visual-Sonics, Toronto, Canada). Anesthetized mice were placed in a supine position on the heating imaging platform with their knees flexed. Ultrasound examinations were performed using both B mode and color Doppler mode, with the 550s scan head, 40–50 MHz probes at following setting: wall filter =  3 mm/s, scan speed =  2 mm/s, dynamic range =  65.0 dB, the number of pulses to radiofrequency cycles =  2, the pulse repetition frequency =  6kHz. A standardized scan was performed according to the “Guidelines for musculoskeletal ultrasound in rheumatology” to demonstrate a typical hypoechoic region representing the joint effusion and synovitis, which corresponds to the intra-articular region. The vascularity near and inside the joint capsule was displayed by a color Doppler mode. After the 2D images were obtained in real time, the images were analyzed and measurements manually determined and calculated using the VevoLab software measurement package. The Surface software were then used to construct the scans into a 3D image, so that the joint space and color Doppler volume can be accurately qualified. The orientation of the ankle and knee joint space of ultrasound image and histology were shown in Fig 1. The ankle joint space was defined as the elongated-shaped region between the tibia and astragalus bones, and the knee joint space was defined as the triangular region between tibia and femur bones.

### Micro-CT analysis

Four weeks after booster immunization, twenty-one CIA mice and seven non-immunized mice were sacrificed. The knee and ankle joints of all mice were analyzed with a μCT80 scanner (Scanco, Switzerland). Three-dimensional reconstructions of knee and ankle joints from these mice were performed using the Micro-CT software. Bone destruction scores were attributed from the micro-CT analysis: 0 = no damage; 1–3 = slight to severe.

### Histological assessment of arthritis

After the Micro-CT scanning, the knee and ankle joints of all mice were collected and fixed, decalcified, and embedded in paraffin. Sections of 5μm thickness were stained with hematoxylin and eosin (H & E). Histological score was graded on a scale of 0–3 by two blinded independent observers for changes in synovial inflammation, pannus formation, and destruction of bone and cartilage. Briefly, 0 = normal, 1 = mild infiltration of inflammatory cells, 2 = moderate synovial hyperplasia and pannus formation, and 3 = severe synovial hyperplasia and pannus formation, bone erosion and destruction.

### Statistical analysis

All statistics were performed using GraphPad Prism 6 (GraphPad, La Jolla, CA, USA). For correlation, we used the Spearman’s test.

## Results

### Quantification of the progression and severity in mouse CIA model by 3D volumetric US

**Fig 2:**
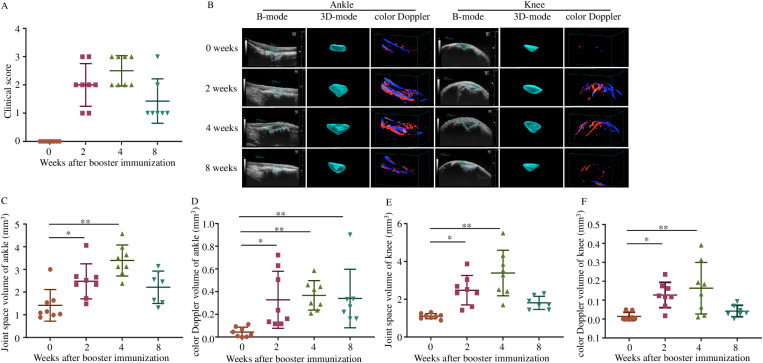
Quantification of the progression in mouse CIA model by 3D volumetric US. 8 mice underwent US imaging of their knee and ankle joints at 0, 2, 4, 8 weeks after the booster immunization. The arthritis severity of all mice was rated at each time point (A), and then the joints were monitored by US both in B-mode and color Doppler mode (B, C). Quantification of volumetric changes of joint space and vascularity at each time point (D-F).

To validate the role of the 3D volumetric ultrasound and color Doppler micro-imaging on the progression and severity of joint inflammation in CIA mice, the same ankle and knee joints of 8 mice were examined during CIA progression. Clinical score is the most convenient and efficient indicator to determine the progress of inflammation in CIA. As shown in Fig 2A, the arthritis symptoms of the CIA mice began to appear after booster immunization, and the clinical score reached a peak at 2–4 weeks and then decreased. Meanwhile, the joint space and vascularity volume on the ankle and knee were also significantly increased as compared to the baseline, reaching a high point at weeks 2–4, and then decreasing (Fig 2B–2F). The results of ultrasound and color Doppler on knee and ankle joints were consistent with the performance of the clinical score.

**Fig 3:**
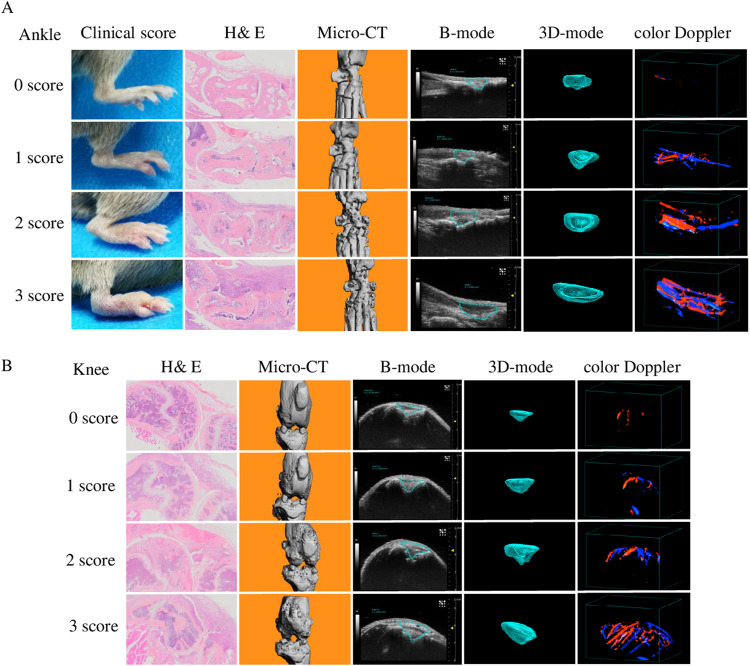
Quantification of the severity in mouse CIA model by 3D volumetric US. Four weeks after booster immunization, US imaging was performed on the knee and ankle joints of 28 mice, and the result were analyzed using the VevoLab software. After that, all mice were sacrificed and the knee and ankle joints were taken for histological and micro-CT examination. Representative images of clinical score, histological score, micro-CT, 2D-B-mode, 3D reconstruction of the joint space and vascular color Doppler signal of the ankle(A) and the knee joints(B).

To further demonstrate the feasibility of 3D volumetric ultrasound and color Doppler micro-imaging in detecting the disease severity of the CIA model, we performed ultrasound and color Doppler, Micro-CT and histology analysis on 21 CIA mice and 7 normal mice. Four weeks after booster immunization, the ankle and knee joints of 28 mice underwent 3D-US imaging and analyzed using VevoLab software. Then the joints were harvested for micro-CT and histology analysis. We found that compared to the normal mice, both the joint space and color Doppler volume of the ankle and knee joints were significantly increased (Fig 3A and 3B). Mice with higher clinical score had larger joint space and color Doppler volume on the ankle and knee joints (S1 Fig). This consistency also applied to histological and bone destruction score (S2 and S3 Figs). Micro-CT results showed that the higher the clinical score of CIA mice, the lower the bone volume of the tibia and talus. Similarly, the more severe the histological and bone destruction score of the knee and ankle, the lower the bone volume of the tibia and talus (S4 Fig). Like the Micro-CT, 3D-US can be used for the detection of disease severity in CIA models.

### Correlation analysis of joint space and color Doppler volume with clinical, histological and bone destruction score

In the CIA model, the clinical, histological and bone destruction score are the most commonly used to assess the progression and severity of arthritis [31,33]. Among these evaluation metrics, histopathological assessment is considered the most objective indicator. It enables the evaluation of synovial inflammation, pannus formation, and the extent of bone and cartilage destruction in CIA mice, thereby providing insights into the progression and severity of arthritis. To determine if the changes of joint space and vascularity that are detected by the 3D volumetric ultrasound and color Doppler techniques correlate with the clinical, histological and bone destruction score, we sought to determine the correlation between them and those scoring system.

**Fig 4:**
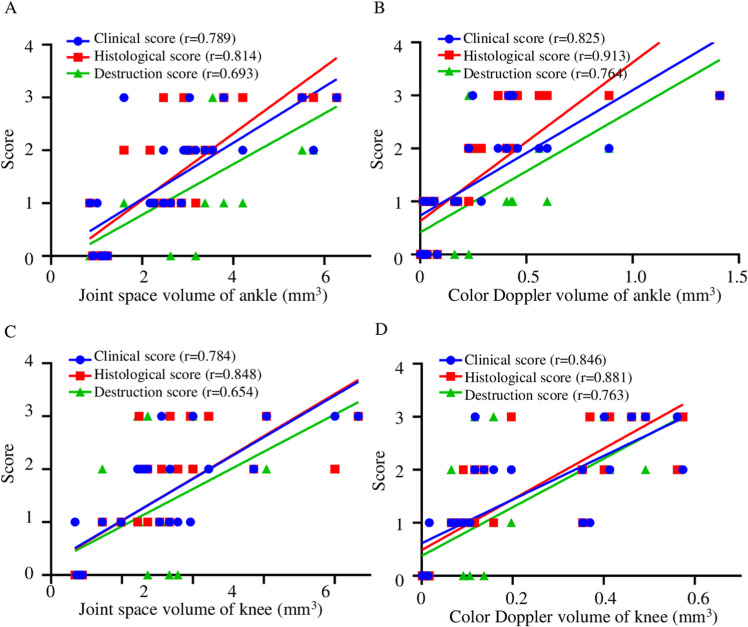
Quantification of joint space and color Doppler volume determined by 3D volumetric US and their correlation with clinical, histological and bone destruction score. Correlation analysis of the joints space volume at ankle (A) and knee (C) with clinical score, histological score and bone destruction score. Correlation analysis of color Doppler volume at ankle (B) and knee (D) with clinical score, histological score and bone destruction score.

At the onset of the disease, the CIA mice exhibited normal ankle and knee joints. As the disease progressed, the clinical, histological, and bone destruction scores demonstrated a gradual increase. Concurrently, 3D volumetric ultrasound and color Doppler imaging revealed a progressive enlargement in joint space volume and vascular volume (Fig 4A and 4B). Notably, a higher histopathological score, indicative of more severe disease, was associated with a larger vascular volume within the joint space.

Importantly, we found a positive correlation between clinical, histological, bone destruction score and joint space volume, color Doppler volume in the ankle and knee joints (Fig 4C and 4D). According to the results of correlation analysis, the joint space volume and color Doppler volume could accurately reflect the severity of joint inflammation in CIA mice.

## Discussion

In the past few years, the volumetric probe has been used in high-end US equipment because of its ability to emit US beams in a volumetric manner [34]. The US images produced in this manner enables the anatomical region to be three-dimensional (3D) reconstructed for evaluating in the longitudinal, transverse, and coronal planes. Therefore, 3D-US technology is an emerging technology that will be used more and more widely in various fields of clinical and scientific research. Because of its three-dimensional reconstruction characteristics, it can more accurately and directly evaluate anatomical structures from different dimensions. Compared with the two-dimensional (2D) technique, 3D volumetric ultrasonography technique is less dependent on the skill of the operator and more reliable in assessment of blood flow and structural changes inside the inflamed joints [35].

CIA model is widely used to evaluate the pathological, pharmacodynamic and pharmacological mechanisms of RA, and can simulate the acute and chronic stages of RA [31–33,36,37]. We can evaluate the severity of ankle synovitis by visual examination of ankle swelling and histopathology. But clinical score has a certain degree of subjectivity, and the histopathological score cannot be used to longitudinal study the pathogenesis of arthritis in the same animal. Therefore, it is necessary to develop new evaluation method that can more accurately and longitudinally evaluate the joints of the CIA model.

Previously, a grey scale of US findings or Doppler signal intensity has been used in CIA mouse model [14], but which is limited by the fact that only semiqualitative data can be obtained. Therefore, accurate and quantitative volumetric changes of joint space and vascularity is needed for the evaluation of disease severity in CIA mouse model.

In the present study and in our prior investigations [38,39], we used 3D High-Frequency ultrasonography and Doppler techniques to quantify the volumetric changes of joint space and vascularity in the ankle and knee of the mice with or without CIA, and then compared the volumetric results with our clinical, histological and bone destruction score. Our results show that the 3D micro-US imaging is a viable, efficient and sensitive method to obtain a detailed 3D data about the joint space and vascularity of arthritis, which is able to accurately quantify synovial inflammation by measuring the increased joint space volume and blood flow in CIA ankles and knees. Moreover, the 3D model-based volumetric changes of joint space and vascularity have good correlation with clinical, histological and bone destruction score, which can be used to provide quantitative data in tracking and classifying the progression and severity of the disease.

The conventional method for detecting and evaluating CIA model by ultrasound has many limitations in reliability [14]. In particular, when traditional evaluation is carried out, the observers must first assess the image and then make the estimation of the score, both of which are highly subjective. In our study, we used the 3D volumetric ultrasound and color Doppler to detect the CIA model. We found that the 3D mode results were superior to the 2D mode in terms of correlation with clinical, histological and bone destruction score, which was consistent with the previous studies [14,40]. In future CIA studies, the use of 3D volumetric US may reduce the waste of experimental animals while improving the interobserver reliability.

However, there are still some limitations, such as low resolution, difficult to detect small lesions; The penetration depth is limited and it is difficult to evaluate deep or facet joints. The image quality depends on the operator’s experience, which may lead to inconsistent results. It has limited transmission to gas and bone and cannot be used for lung and bone examination. Nonetheless, 3D volumetric US is an important non-invasive tool suitable for real-time monitoring and initial evaluation.

In summary, 3D volumetric ultrasound and color Doppler is a cost-effective and non-invasive method to detect and quantify the volumetric changes of joint space and vascularity, which is a sensitive and reliable imaging technique for assessing inflammatory activity and joint damage during the progression of CIA.

## Conclusions

This study demonstrated the noninvasive and reproducible detection of pathological changes associated with arthritis in CIA mice using 3D volumetric ultrasound and color Doppler imaging. These modalities effectively assessed disease activity and progression, as well as detected bone erosion and soft tissue changes. The quantitative features of 3D volumetric ultrasound and color Doppler imaging enhanced diagnostic accuracy. Collectively, these imaging techniques provided a more sensitive and accurate approach for the early diagnosis and evaluation of RA, treatment monitoring, and prognostic assessment. Despite certain limitations, these methods significantly augmented our capacity to monitor disease progression, evaluate therapeutic interventions, and reduce reliance on invasive procedures. Future advancements in imaging technology and standardization are expected to further enhance their utility in arthritis research.

## Supporting information

S1 Fig Mice with higher clinical score had larger joint space and color Doppler volume on the ankle(A-B) and knee joints(C-D). (TIF)

S2 Fig Mice with higher histological score had larger joint space and color Doppler volume on the ankle(A-B) and knee joints(C-D). (TIF)

S3 Fig Mice with higher bone destruction score had larger joint space and color Doppler volume on the ankle(A-B) and knee joints(C-D). (TIF)

S4 Fig The higher the clinical score(A-B), histological score(C-D) and bone destruction score(E-F) of CIA mice, the lower the bone volume of the tibia and talus. (TIF)

S5 File This Supplemental File has six sheets that contain raw data of Figs 2, 4 and S1–S4. (XLSX)
