# Supplementary figures and images for: In vivo quantification of collagen-induced arthritis mouse model by three-dimensional volumetric ultrasound and color Doppler
Source: PLoS One. 2025 Apr 7;20(4):e0321124. doi: 10.1371/journal.pone.0321124 (PMC11975066; doi:10.1371/journal.pone.0321124)

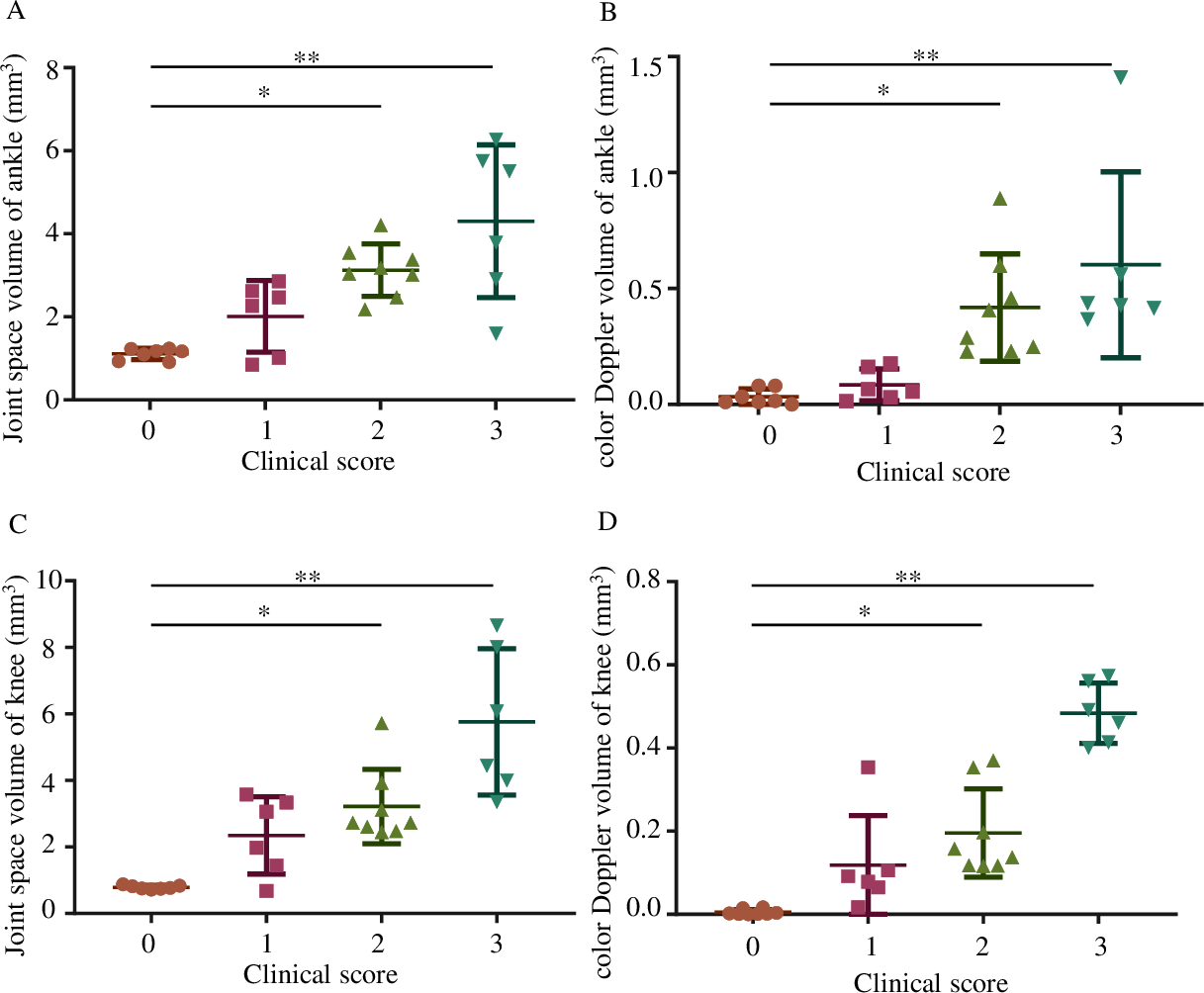

Supplement: S1 Fig — (TIF) [file pone.0321124.s001.tif]

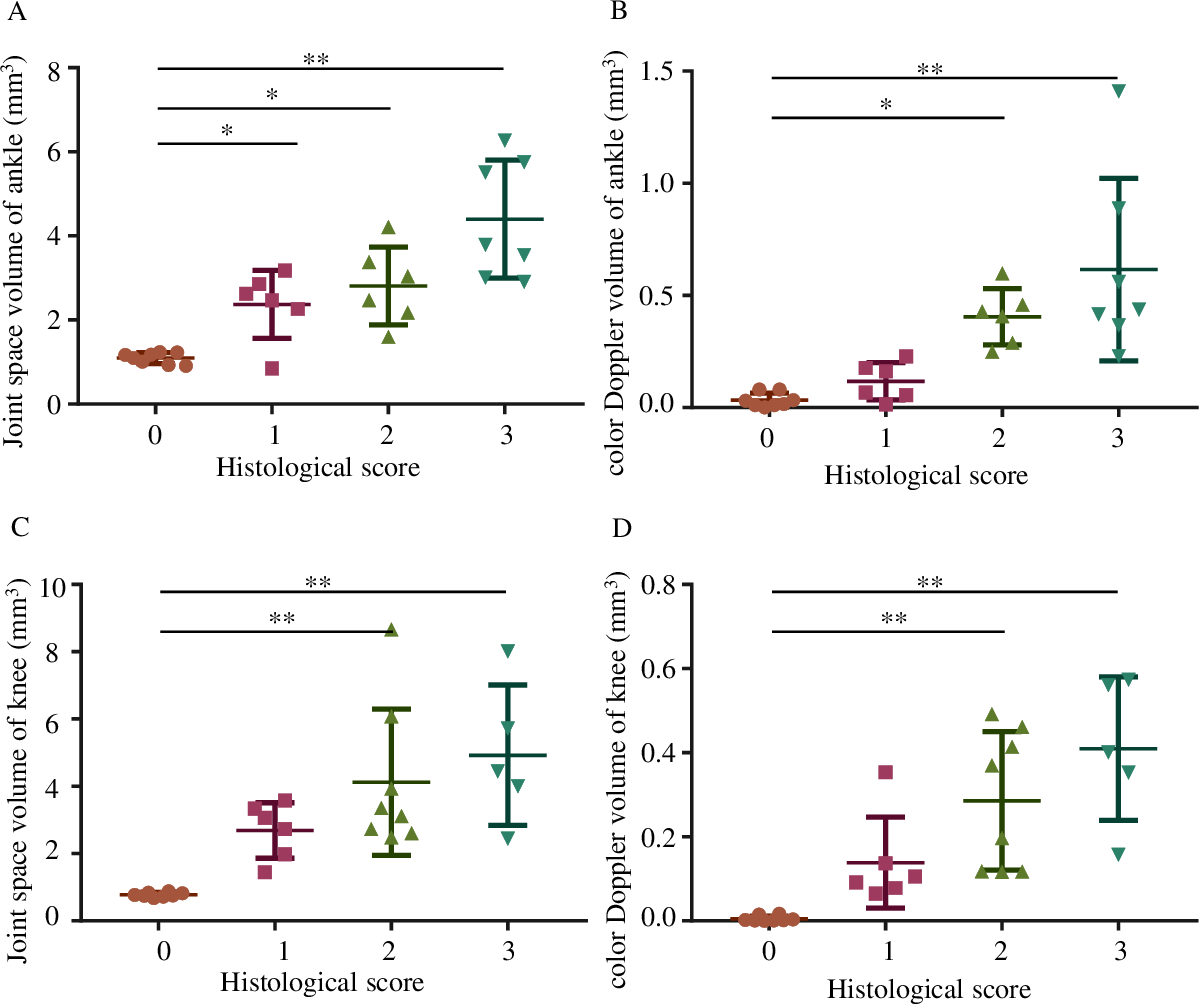

Supplement: S2 Fig — (TIF) [file pone.0321124.s002.tif]

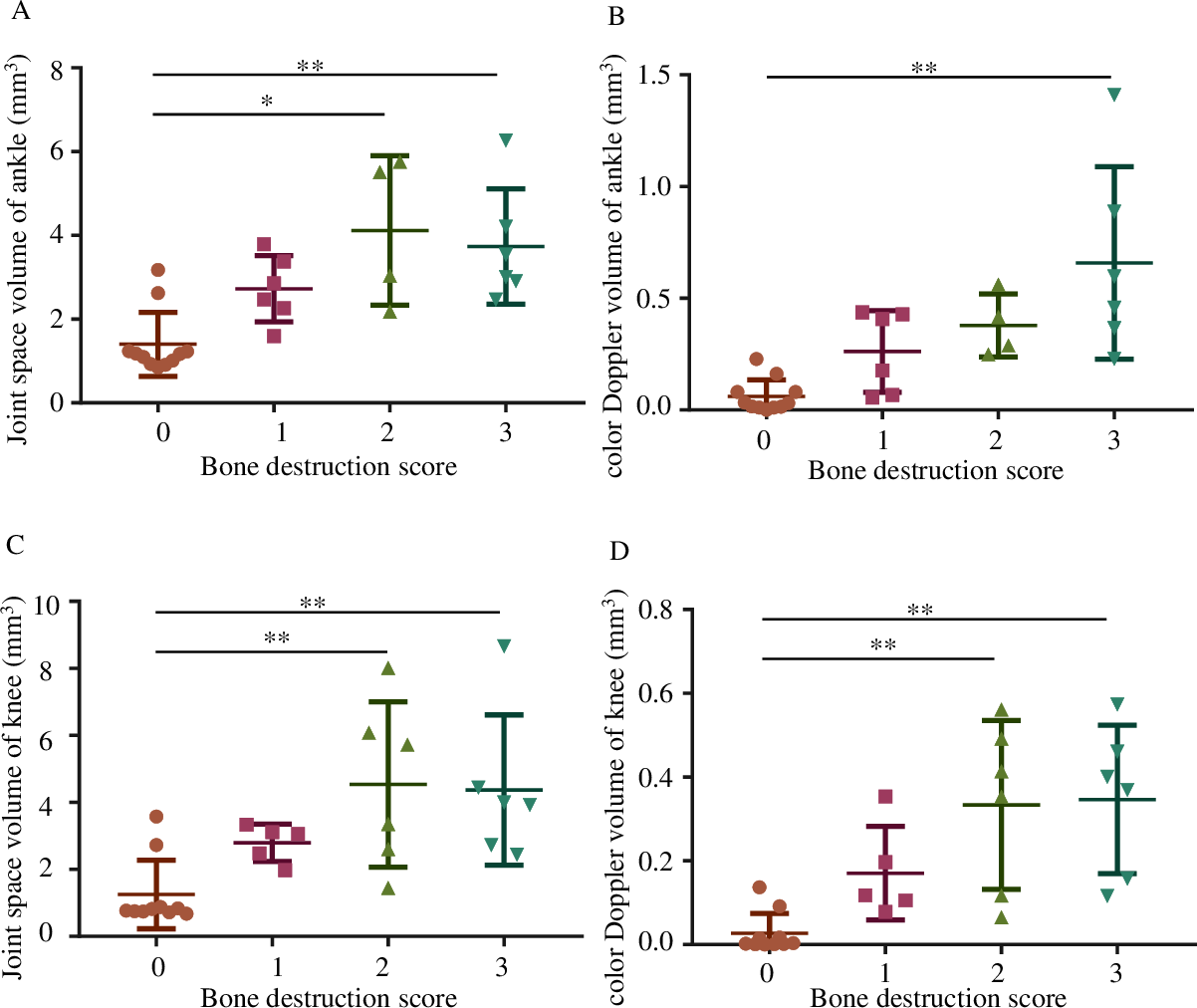

Supplement: S3 Fig — (TIF) [file pone.0321124.s003.tif]

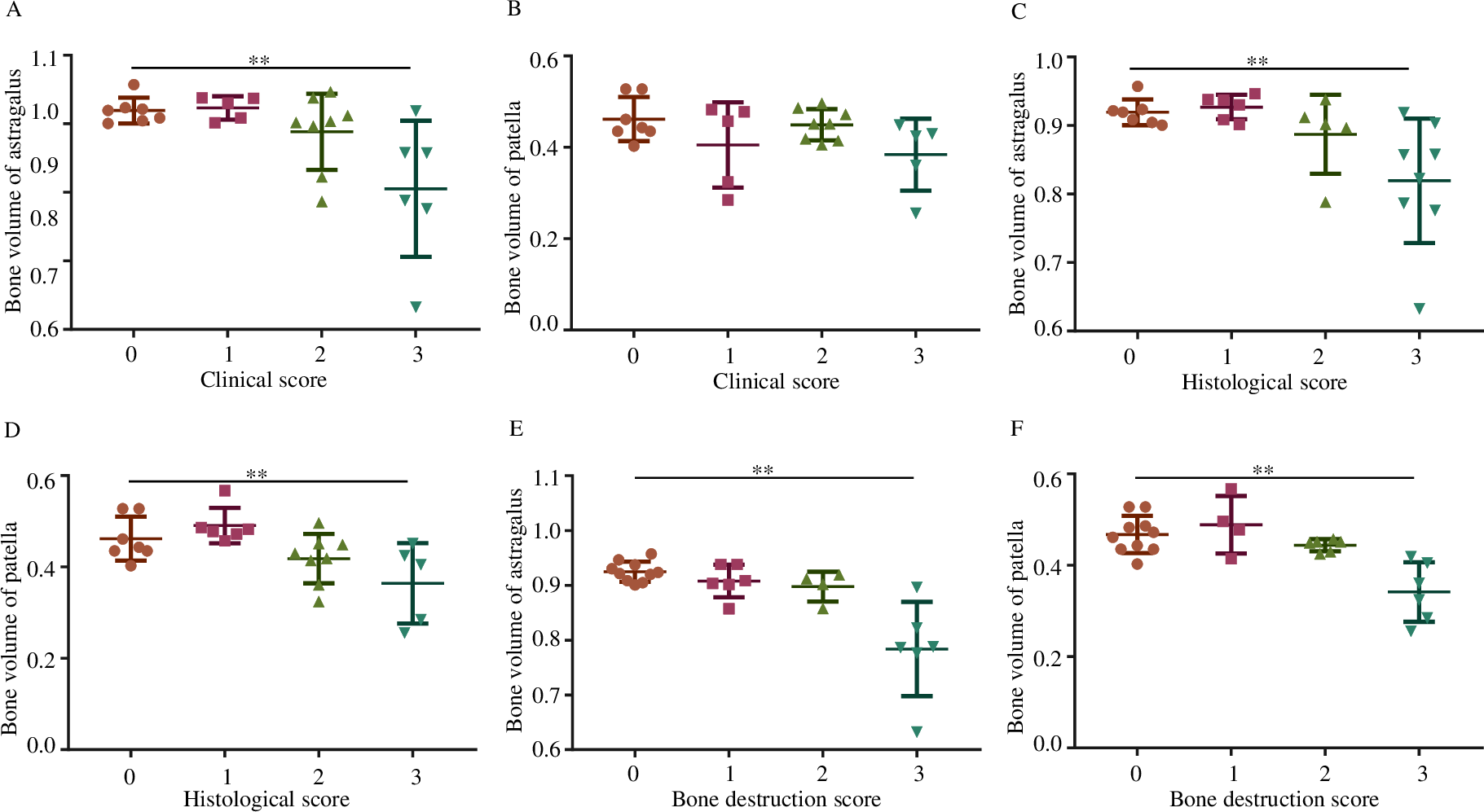

Supplement: S4 Fig — (TIF) [file pone.0321124.s004.tif]
